# The Decay of the Teeth

**Published:** 1897-03

**Authors:** 


### Abstracts and Translations.

### THE DECAY OF THE TEETH.[1]

If the story told us by the prehistoric remains in our museums is to be relied upon, we may be sure that decay in human teeth has existed as long as the race itself. From the earliest crania we see evidences of carious teeth, and we may consider it to have been as extensive as most human ills. At the present time decay is perhaps as prevalent as ever before, caused mainly by the unnatural manner of living. Luxuries, mixed and adulterated foods, containing starch and sugar in excess, have much to do with it, for they are the principal ferment producers. The acids formed by these ferments decalcify the tooth structure, thus giving entrance to organisms, always present in the mouth, and these continue the work of destruction. The various views concerning the nature of decay of the teeth have undergone manifold changes in the course of the centuries which have elapsed, and it has waited for this,—the era of bacteriological investigation,—which has given to us facts, that makes its nature visible to all. So far back as the time of Hippocrates (450 B.C.) it is stated that the cause of decay was probably "the bad juices caused by stagnation." Galen and others state that trouble with food, lack of nourishment, makes the teeth weaker, and also say that the excess of food makes a sort of combustion, which causes decay. Then we had the inflammation theory, according to which, by means of an inflammatory process, first the lime salts of the tooth are dissolved and then the remaining basis substance liquefied. Such a theory is even to-day defended by some writers, in spite of the fact that the results of more recent investigators show it to be untenable. This theory is sheer nonsense, for there are, in every inflammation, elements which do not appear in the decaying process of tooth-tissue, and the cardinal symptoms of inflammation are not observed in dental decay. The causes and materials which always cause inflammation will not produce it in the

---

[1] A condensed report of a paper written by Professor Jung, of the University of Berlin, the translation by Samuel C. Prescott, of Cambridge, Mass. Prepared and read by request before the Harvard Odontological Society by R. R. Andrews, D.D.S., Cambridge, Mass.

matrix substance of the dentine, and then decay takes place in dead teeth as well as in living ones. Inflammation is a vital process. It can only be considered in living tissue. One can produce artificial decay in dead teeth, even outside the mouth, differing in no way from the natural decay, and this is a proof that external agents alone, without vital reaction of the tissues, are enabled to produce the complex symptoms of the disease. Representatives of the inflammation theory to-day are Heitzmann and Bödecker. They claim to have microscopically proved that a genuine inflammation of the dentine of the tooth is present very extensively even in places which have no connection with the tooth-pulp or the pericementum. Through the inflammation the lime salts are dissolved, and the matrix or basis substance of the dentine liquefies, and there results a cavity, which may be filled with "medullary corpuscles;" if these latter fall to pieces, there results an abscess in the root of the tooth. If they take up lime salts again, then a *restitutio ad integrum* takes place. It is strange that these pictures which Heitzmann and Bödecker have seen and also drawn could be confirmed by none of the many investigators of the decay of the teeth up to the present time. Another theory of decay we may call the worm theory, according to which small worms might destroy (devour) the substance of the teeth. This view was for a long time the current one among the earliest writers. Then there was the fermentation theory. Remnants of food fermenting between the teeth cause the teeth themselves to ferment, but it has been proven that the alkaline products of fermentation cannot attack a tooth. Then there was the chemical theory. This has followers, who consider that decay takes place as a result of inorganic acids, and those who consider organic acids as the cause of destruction of the tooth-tissue. But such inorganic acids are not present in the mouth. Organic acids, however, will destroy the teeth, so that macroscopically we are presented with a picture similar to the real decay, but which, by microscopical research, appears entirely different. The chemical theory is one which has obtained longest, even up to recent times. Thus, Magitot, Wedl, Tomes, Taft, Schlenker, and Baume, among the authors of the last three decades, were late followers of this theory. The chief part of the decomposition, besides that of the acids brought into the mouth in food and drink, is ascribed to the acid mucus and saliva, especially by digestive disturbances, wasted secretions, acid decomposition products, etc. Another theory was the parasitic theory, according to which parasites may be promoters of decay. Thus were Leeuwenhoek's "animalcula" accused in this direction (not by Leeuwenhoek himself, however). Erdl found a "parasitic vegetable;" Ficinus, infusoria, which he designated by the genus name of "denticola;" Klencke, his "zahn-thierchen." The parasitic theory gained many advocates by the publication of the researches of Leber and Rottenstein. To lepto-thrix buccalis these authors conferred a particularly active *rôle* in the work of decomposition. The "elements" of the fungi present in every oral cavity multiply in the small tooth-canals in vast numbers, distending them, and thereby affording an entrance of acid into the depths, thus leading to the decomposition of the tissues. Indeed, it was believed that this fungus itself could bore through enamel, separate its prisms from one another, and then break it to pieces. The new theory was not at first generally accepted. It has continually gained ground, and has done much to prepare the way for the chemico-parasitic theory of Miller. Another theory was the electrolytic theory. In this the crowns of the teeth were electro-positive (Bridgman, Chase, and others); the dentine, electro-negative; and this, by the addition of moisture, produces an electrolytic dissociation of the mouth fluids, and the acids separated would result in decalcifying the tooth at the positive poles (the crowns). Since the tooth-tissues are non-conductors this theory is not at all tenable, as for the generation of an electric current both excitants must be conductors. There were various other factors given as causes of decay,—mechanical injuries, fissures resulting from change of temperature, acid foods, excessive use of sugar, etc.,—until the researches of Miller and his chemical parasitic theory brought to us the light and truth. Miller gives the results of his researches in the following statements: The decay of the teeth is a chemico-parasitic process, consisting of two significantly marked steps,—the decalcifying or softening of the tissues and the dissolving of the softened residue. The decalcification of the enamel means the total destruction of it. The source of the acids necessary for the softening of the tissue is not difficult to determine; they are the remnants of food containing starch and sugar retained in the cavities (fissures in the enamel, narrow cracks between the teeth, etc.), and which by fermentation form acids. The second step—the dissolving of the softened tooth-substance—is accomplished by fungi always found there. We have seen that many of the mouth fungi possess the power of dissolving or peptonizing albumen or albuminoid substances,—*i.e.*, to change them into a soluble modification. We know that the dentine matrix consists of an albuminoid substance. Thus we have the explanation of the second step of tooth caries, especially as solution of the decalcified dentine by fungi may be directly proved and experimentally observed. Consequently, Miller lays special stress on the action of ferment acids arising in the cavity, while hitherto only the acids brought in from without have been regarded as active in the process of decalcification.

Miller's theory is to-day recognized by the great majority of all authorities on dental science. There are only a few who believe in the theories of the last decade who uphold the formerly current combustion theory of the pure chemical theory, and who contest the work of Miller under the assumption that the presence of micro-organisms was purely accidental and had nothing whatever to do with the disease itself. Thus, Baume, especially in the newer editions of his "Lehrbuch," holds to the opinion that the fungi might luxuriate in the tissue undergoing destruction in the manner in which fungus elements luxuriate in decaying tissues. The bacterial fungi must be, therefore, the result of caries. Baume assumes four steps in caries: 1, the dentine becomes transparent; 2, turbidity of the transparent dentine, as the result of softening; 3, formation of pigment and great softening; 4, cartilaginous dissolving and destruction. He states that only in the fourth step are fungi present.

As an advocate of the pure chemical theory, Baume seeks to prove that the acids alone may be sufficient to carry on the work of destruction; in reality the characteristic microscopical changes in activity in caries cannot be explained as derived from the action of such acids. The picture under the microscope makes the presence of micro organisms absolutely necessary.

It may be proper to consider these changes at some length. There is observed in the enamel at the places where caries is taking place, first a roughness; the enamel has a whitish, cloudy appearance; further, a softening of the enamel follows, and accompanying this complete disappearance of the same. It is decomposed to a white powder and a cavity results, the edge of which is more or less colored brown. In general this decomposition of enamel goes on comparatively slowly up to the point when its whole thickness is destroyed, even to the dentine. In the dentine the process goes on more readily and also expands laterally, and, as a result, the enamel is attacked from the inside and broken down. The dentine is not like the enamel. It is not entirely destroyed by the decalcification, but there remains behind a cartilaginous mass,—the decalcified basis-substance of the dentine (tooth cartilage). According to the rapidity with which the carious process continues, the part attacked takes on a diversified coloration (pigmentation),—that is, it remains white in the rapid form, but is light brown or black when the process has a more or less pronounced chronic course.

Microscopically, we see only the coloration of the edge in the enamel. In the dentine the canals appear greatly distended and filled with fungus matter, micrococci, bacilli, etc.; in some cases a solution of the substance between the canals takes place, whereby neighboring canals fuse with each other, and thus numerous small cavities result, which, merging into each other, soon settles the destruction of the tissue. The deeper layers of the decalcified tooth bone (dentine) are not so full of bacteria; they are only seen here and there, so we may assume that the entrance of these proceeds with the dissolving of the tissues.

The decalcifying can naturally be accomplished by acids only, and on this account we may designate the decalcification as the first and the entrance of bacteria as the second step in caries. Caries of the cement-substance sometimes takes place on exposed roots, and then appears as a softening of the tissues and subsequent cavities, which are found to be mostly without distinct edge or great depth. The decalcification and solution then goes on as in dentine. The canals of the cement become infiltrated with bacteria and broadened, especially the fibres of Sharpey and the cement lacunæ. The course of decay beyond this point is the same as with dentine,—that is, after the disappearance of the walls of the canals the cavities join, a greater cavity results, and the dissolution of the tissue is made complete.

According to Miller the following are observed only as accompanying phenomena in caries: the transparency and pigmentation of the dentine, phenomena which have been earlier observed as characteristic of caries. (Baume and others.) If one looks at a section of a tooth in which caries is present in the first stages, he will see a conical transparent part in the dentine whose apex extends in the direction of the canals nearly to the pulp, while the base of the carious place lies on the surface. This transparent zone of dentine is not observed in dead teeth. It is present without the phenomena of caries in parts of the dentine upon which a chronic irritation exists, as, for example, the cutting surfaces that become deeply worn are subject to an irritation of a chemical or thermal nature to the pulp, and, as a result, we find there has been formed a transparent zone. Such a transparent zone sometimes appears in the dentine of the root. We must, therefore, conceive the transparency to be a vital process, by which, in consequence of an external irritation (especially acids), the tooth-canals in the dentine are induced to an increased laying up of lime salts, so that as the outer surface of the canals change (calcified) these become narrower and the lumen of the canal is lessened. (Miller.) Under these conditions the dentine becomes more uniform, since it now consists only of hard tissues and not, as in the normal condition of hard and soft tissues, alternating with one another. The homogeneity necessitates another index refraction of the light rays: hence the transparent zone.

The views concerning the nature of this phenomenon are still somewhat unsettled. While Tomes, Magitot, Walkhoff, and others stand out for the assumption of a partial calcification of the dentine canals, it is doubted by Wedl and others. Leber and Rottenstein, as well as Schlenker, seek the transparency in a partial decalcification of the dentine. According to Baume, it is produced by an obliteration of the canals, which may be caused by growth of basis-substance (dentine matrix). Wellauer puts forth the view that the transparency consists of a partial withdrawal of the lime contents of the matrix, either through periodical infiltration of the dentine canals with lime salts in solution, or by partial or complete calcification of the contents of the canals.

According to the views of some writers (Black, etc.), the pigmentation of carious tissue may be caused by the action of sulphur compounds, which may be formed by fermentation processes; according to others, by direct coloring substances, as coffee, tobacco, etc. Pigment-forming bacteria are also accused in this direction. We may accept Miller's view that bacterial processes play the principal part here; as every other organic substance which is decomposed by bacteria in course of time assumes a dark color, the tooth-tissue does the same. The processes are supported by oxidation processes, for which the oxygen of the air is ready for disposition in plentiful amounts. Pigmentation always takes place also in sound dentine if this is exposed. We see it in the wearing away of the enamel on the grinding surfaces. The question now arises, Of what nature are the bacteria of caries? After Leber and Rottenstein the chief advocates of leptothrix buccalis, Clark, Underwood, and Milles, have been engaged with the study of these organisms. Clark describes his dental bacteria, which he found in carious teeth, as follows: They can easily be confounded with the vibrio regula or with the forms described by Cohn under the genus name of Spirochætæ or with spirochætæ plicatilis; they have a hardly noticeable screw-like motion and,

I believe, various forms. Their activity is heightened by the addition of acid. Without this they appear sleepy and lifeless. They are found nowhere in sound dentine, but sometimes in the deposit on the teeth. Vibrio regula and spirochætæ plicatilis, on the other hand, are never found in the canals of the carious teeth, which circumstance alone separates the dental bacteria from both these kinds. Miller could not confirm the results of Clark. He wrote concerning them: I have not found in the dentine canals the bacteria described by Clark, to which he ascribes the leading part in caries. This form occurs especially on the tooth and not in the cavities. The vibrios cannot exist in the canals. Underwood and Milles reported in 1881 that they had found various forms of micro-organisms, especially micrococci, small rod forms, and oval bacteria, short bacilli present in carious dentine; so, like the other inquirers of that time, they confined themselves to proving views concerning the nature of the micro-organisms involved by microscopical examination of the fungus masses contained in the dentine canals. Miller opposed the theory of leptothrix buccalis by the theory of a specific micro-organism of the mouth cavity which might produce all other diseases besides caries. He proved that the formations which Hallier, and after him others, had accepted as elements of leptothrix buccalis, which was represented as a fungus, could not exist in connection with this bacterium; that what had been considered as swarmspores of leptothrix buccalis, motile fission fungi of certain kinds, and that which had appeared as swarmspores in the resting stage, were cocci, as they are present in the mouth cavity in manifold forms.

It appeared that the iodine reaction described by Leber and Rottenstein could not be considered as characteristic of leptothrix, since Miller could prove that a variety of kinds of filiform fungi are present in the mouth, which show a beautiful violet color with iodine and acids, and, moreover, that these filiform fungi, which give the iodine reaction, are quite clear and regularly jointed, while leptothrix is described as thin, long, unjointed threads.

The leptothrix appears to play only a secondary part in caries, according to the pre-ent ideas, since growth of leptothrix appears only on the layers of carious dentine nearest the surface (mostly decomposing), while the threads are not met with in deeper layers. From the deeper layers of carious dentine Miller was able (in 1880) to isolate five kinds of bacteria, to which, in view of the place where they were found, a close primary relation to carious processes could be ascribed. The first of these forms that he found were cocci and diplococci, found either simply or in chains; the second has several forms,—cocci, bacteria, bacilli, and threads. The third form, very small abundant irregular cocci, seldom joined in chains. The fourth also occurs in the form of cocci, which show an extraordinary diversity of size. The fifth is morphologically most interesting of all. It occurs in short rods, with all variations from straight to semicircular; it is also found joined in twos, forming a small S; also as threads, but more or less like spirilla, jointed or unjointed. All these kinds seem to have the general property to set in fermentation solutions of fermentable carbohydrates, whereby lactic acid is produced; and this acid has a very strong affinity for the lime in tooth-tissues.

By combination of the results which Miller has obtained concerning the properties of these five caries fungi and some twenty other mouth bacteria, he has formulated his view of the subject of the activity of these forms in regard to caries. Of the twenty-five kinds, sixteen were proved as able to form acids by fermentation of the carbohydrates. These might, therefore, come into the consideration of the first step in caries. The majority of all showed peptonizing qualities, which they would be able to use to dissolve the decalcified dentine and cement. Only those were considered as caries bacteria which were proved active in carious dentine.

After Miller, Gallippe and Vignal have been engaged to some extent with the study of caries bacteria, and they succeeded in isolating six different kinds. Four of these occurred in all the eighteen teeth investigated, one kind only eight times, and one only five times. Like Miller, Galippe and Vignal had in their studies used only gelatin beside the liquid media in studying the life history of these organisms. More recently Professor Jung, of the University of Berlin, carried the experiments further by using solid media, which permitted treatment at the temperature of the mouth cavity.

Investigating in this way with seventy-two teeth, Professor Jung succeeded in isolating ten different kinds of bacteria from carious dentine. Besides these ten kinds, an eleventh was found; however, not constantly, and he is not sure that it is an active caries fungus. There were always several kinds present in carious dentine at the same time, and we may consider it as settled that the decay of teeth is not due to a specific bacterium, but to a mixture of infection.

In dead teeth caries takes place with exactly the same phenomena as in living teeth. Caries may also be produced in artificial ways, differing in no way from the genuine caries.

Miller has proved that the phenomena of caries in animals are exactly like those in the human teeth.

There are only a few now who do not believe in Miller's theory. All recent authorities are working after Miller's ideas, and we may assume that questions concerning the nature of decay of the teeth have now found their final solution. There are other secondary phenomena, as revealed by accurate microscopical examination of carious teeth, that await a satisfactory explanation, as, for instance, the thickening of the so-called Neumann's sheaths, the appearance of the so-called pipe-stem formations, and the presence of granular elements (not vegetable) in the canals of the dentine.

The thickening of the so-called Neumann's sheaths in decayed dentine may, according to Neumann's assertion, often be so complete that the lumen may entirely disappear. Tomes allows only a partial obliteration, and Leber and Rottenstein dispute this, although they do not know how to maintain the fixed facts in regard to the thickening of the walls. Miller explains the phenomenon by the pressure of the collected bacteria masses in the canals, by means of which a compression of the wall-sheaths is brought about. That it cannot deal with vital processes is made clear from the circumstance that the phenomenon is also observed in dead teeth.

The presence of rows of shining irregular globules in the canals of carious dentine in early stages is considered by many as a vital phenomenon,—an attempt of the pulp to oppose the progress of caries. The facts opposed to this are the appearance also observed in dead teeth. Tomes, Magitot, and others considered the formation as lime granules; Wedl, Black, and others as fat drops. The investigation leaves the field still open. Something not yet understood is the question relating to the so-called healing of caries, that rare phenomenon in which the decaying process comes to a stand-still, and the carious, weakened dentine becomes hard again without any addition.

These are the facts about the primal causes of dental decay. The question, "What are the predisposing causes?" yet remains to be answered, and it is a field for much thought. It is not wholly hereditary, or faulty formation, that causes the dentine to become a culture medium for the fungi of the mouth. We know there are constitutional factors at work, retarding the healthy natural vigor of the system, weakening the dental tissues as well. Women about to become mothers, people attacked by constitutional disease, overworked students in our schools and colleges, all these suffer much at such times from decaying teeth. A return to health retards and will sometimes stay the trouble. Are we not all familiar with a class of patients that have had a fine set of teeth up to the fiftieth or sixtieth year, who suddenly find their teeth troubling them and rapidly decaying? In such cases, in my own experience, and I remember several, it seemed impossible to arrest the trouble, and in two cases death followed in a short time. Impaired vitality from whatever cause has a very marked effect on the dentine, and under its influence it rapidly becomes a culture medium in which the caries fungi thrive; on the other hand, the exposure of the dentine from wear or from a break does not always end in decay. In health and vigor decay may become arrested by a natural process. Miller's work is a grand one, but we have much yet to learn. I shall invite your attention now to the lantern exhibit, where we shall see the dental tissues in health and in decay.

